# Biochemical characterization of wood decay and metabolization of phenolic compounds by causal fungi of grapevine trunk diseases

**DOI:** 10.1371/journal.pone.0315412

**Published:** 2025-04-16

**Authors:** Erin R. Galarneau, Christopher M. Wallis, Kendra Baumgartner

**Affiliations:** 1 United States Department of Agriculture-Agricultural Research Service, Plant Genetics Resources Unit, Geneva, New York, United States of America; 2 United States Department of Agriculture-Agricultural Research Service, Crop Diseases, Pests and Genetics Research Unit, Parlier, California, United States of America; 3 United States Department of Agriculture-Agricultural Research Service, Crops Pathology and Genetics Research Unit, Davis, California, United States of America; University of Salento Department of Biological and Environmental Sciences and Technologies: Universita del Salento Dipartimento di Scienze e Tecnologie Biologiche ed Ambientali, ITALY

## Abstract

Grapevine trunk diseases, such as Esca, Botryosphaeria dieback, and Eutypa dieback, are caused by various Ascomycota and Basidiomycota fungi that colonize wood and form internal lesions. Basidiomycota fungi, such as *Fomitiporia* species, are associated only with the trunk disease Esca, and are wood-decay fungi. Variation in the extent of lesion development among the fungal pathogens reflects a combination of fungal virulence and host susceptibility. To evaluate factors that may affect lesion development, we compared *in vitro* wood-decay abilities and tolerance of host secondary metabolites (cell-wall and soluble phenolic compounds) of four fungi that cause trunk diseases: *Eutypa lata* (Eutypa dieback), *Fomitiporia polymorpha* (Esca), and *Diplodia seriata* and *Neofusicoccum parvum* (Botryosphaeria dieback). Fungi were grown on autoclaved blocks of *Vitis vinifera* ‘Merlot’ wood for six months, to examine fungal colonization of wood cells and percentages of wood components remaining after decay. Fungi were also grown on medium amended with starch, pectin, lignin, cellulose, hemicellulose, tannic acid, gallic acid, magnesium sulfate, or grape wood powder, to determine cell wall-degrading enzyme activity and impacts on fungal growth. Lastly, to determine tolerance of phenolic compounds, fungi were grown in medium amended with piceid, rutin, epicatechin, or gallic acid. Our novel findings for *F. polymorpha* include its preferential degradation of hemicellulose and pectin (and detection of corresponding enzymatic activities), but no degradation of lignin, in spite of growth in lignin-amended media and detection of laccase, lignin peroxidase, and peroxidase activities. Together, these findings suggest *F. polymorpha* has characteristics of both brown-rot and white-rot fungi. The type of wood decay caused by *D. seriata* and *N. parvum*, based on their degradation of pectin, cellulose, hemicellulose, and lignin (and detection of corresponding enzymatic activities), is characteristic of a soft rot, similar to that of *E. lata*. Unique among these three Ascomycetes was induction of *N. parvum* growth by piceid, rutin, epicatechin, and gallic acid, and efficient metabolism and/or detoxification of these phenolic compounds by *N. parvum*. As all four fungi metabolize components of the wood as substrate, and also can metabolize/detoxify host-defense compounds, a clearer understanding of their roles as wood-decay fungi might further research on managing the chronic wood infections.

## Introduction

Trunk diseases (Botryosphaeria dieback, Esca, Eutypa dieback, Phomopsis dieback) are among the most widespread and damaging diseases of grapevines worldwide. The fungal pathogens, which span four orders in Phyla Ascomycota and Basidiomycota [1], establish chronic infections in the permanent, woody structure of the vine. Wood infections are typically localized near wounds to the spurs, cordons, or trunk. Such wounds include those made to the vine intentionally, during pruning and training, or are through injuries, made by mechanical-harvest machines or due to freezing temperatures. Symptoms range from stunted growth and death of fruiting positions (‘dieback’ caused by Botryosphaeria-, Eutypa-, and Phomopsis diebacks) to spotted fruit (‘measles’ caused by Esca). When trunk diseases go unmanaged, yield losses accumulate over time, thereby shortening the productive lifespan of vineyards [2,3].

Trunk diseases are difficult to manage. There are few fungicides that effectively protect pruning wounds and they are legally labeled for use in only a few US states. Thiophanate-methyl is effective against Eutypa dieback-pathogen *Eutypa lata* and Botryosphaeria-dieback pathogen *Neofusicoccum parvum*, but not against Esca pathogens *Phaeomoniella chlamydospora* or *Phaeoacremonium minimum* [4,5]. Further, timing applications is imprecise, in part because climate-based models of spore dispersal are available for very few of the pathogens (e.g., one exception is for *P. chlamydospora* [6]). An alternative approach is pruning when wounds are less susceptible, albeit only for *E. lata* [7,8] and *N. parvum* [9]. However, there is no way for growers to evaluate pruning-wound susceptibility in the vineyard. Novel management practices are therefore needed.

The trunk-disease pathogens colonize wood, but they are not all characterized as wood-decay fungi. They vary in the plant cells they colonize, the mechanism of damage, and in their ability to metabolize/detoxify host-defense compounds [10]. Recent work with European Esca pathogen *Fomitiporia mediterranea*, a wood-decay fungus that causes a white rot, reports evidence of non-enzymatic decomposition of lignin, using iron [11]. This discovery opens a new door in disease management, by potentially managing this species (and others with the same lignin-decomposing capability) through applications of iron-chelating compounds [12].

Common to the infection process among the pathogens is the formation of internal ‘wood lesions’ or ‘wood cankers’, which may be due in part to wood decay. The type of wood decay caused by *F. mediterranea* is technically referred to as a ‘white rot’ [13]. Like other white-rot fungi, *F. mediterranea* (previously known as *Phellinus punctata*) decomposes all three main components of the wood (cellulose, hemicellulose, and lignin) [11,14], leaving the wood soft in texture and white/yellow in color [15]. The type of wood decay caused by *E. lata* is technically referred to as a ‘soft rot’ [16], a term currently applied to the type of wood decay caused by Ascomycota fungi [17]. Like other soft-rot fungi, *E. lata* partially decomposes cellulose, hemicellulose, and lignin [18,19], leaving the wood hard in texture and dark in color.

Botryosphaeria-dieback pathogens *N. parvum* and *Diplodia seriata* produce wood-degrading enzymes *in vitro* [20]. Some of these enzymes degrade phenolic compounds, which compromise cell wall-strengthening molecules (e.g., lignin, tannin), and others exist as small, soluble molecules with antibiotic or antioxidant activity [21]. Lesions in wood inoculated with *D. seriata* or *N. parvum* look like that of *E. lata* [22], but the type of wood decay caused by the former two species has not been characterized in chemical or anatomical terms. Such gaps in the knowledge of fungal ecology, especially for these widely distributed species [23], make it difficult to identify host responses associated with resistance, thereby hampering breeding programs. Mixed infections are reported from field studies, based on isolation of multiple pathogens sometimes from the same small piece of wood [e.g., [24]]. The individual wood-decay ability of one species may influence colonization or wood-decay by another [25]. Indeed, Basidiomycete *Fomitiporia polymorpha*, a North American Esca pathogen [13], causes larger lesions when co-inoculated with *P. chlamydospora* [26].

Lesion development is affected by the pathogen’s capacity to overcome, neutralize, or avoid plant defenses. Host plants produce ‘phytoalexins’ (e.g., phenolic compounds), to limit further colonization and aid in cell repair [27,28]. High concentrations of stilbenes, a class of phenolic compounds, in the woody stems of grapevines inoculated with *D. seriata* and *N. parvum* (compared to those of non-inoculated plants) suggests that stilbenes may have a role in the host-defense response to Botryosphaeria dieback [29,30]. From naturally infected grapevines with Esca, high concentrations of stilbenes are reported in symptomatic wood, compared to apparently healthy (‘asymptomatic’) wood from the same vine [31]. High stilbene concentrations have been associated with resistance to *N. parvum* infection in genotypes of *Vitis vinifera* subspecies *sylvestris*, an ancestor of cultivated wine and table grapes [32]. Stilbenes have been found to inhibit the growth of *D. seriata, N. parvum, E. lata, P. chlamydospora*, and *F. mediterranea*, in *in vitro* experiments [20,30], albeit at concentrations often exceeding those reported *in planta* [33]. Nonetheless, such inhibition suggests that stilbenes in the wood may limit colonization, and thus virulent pathogens that colonize wood must overcome, neutralize, or avoid such compounds. Indeed, the ability to degrade or overcome phenolic compounds has evolved among numerous, unrelated taxa of plant-pathogenic fungi [21].

This study aims to characterize the wood-decay abilities of causal fungi of trunk diseases commonly found in California vineyards: *E. lata*, *D. seriata*, *N. parvum*, and *F. polymorpha*. A ‘top-down’ approach was used to determine how the pathogens colonize grape wood, in terms of the breakdown of complex cell-wall polymers (cellulose, lignin, and tannins), and in terms of fungal detoxification/ neutralization of phenolics, at biologically relevant concentrations [33]. Altogether, results should fill gaps in the knowledge of the infection processes, which will in turn inform research on disease management and host resistance.

## Materials and methods

### Fungal isolates

Isolates of *Neofusicoccum parvum* UCD646So [34], *Diplodia seriata* SBen831 [35], *Eutypa lata* Napa209 [36], and *Fomitiporia polymorpha* WFB1 [26] were maintained on Potato Dextrose Agar (PDA; Difco Laboratories, Detroit, MI, USA). Isolates were chosen in part based on their relative virulence *in planta*, as examined in previous experiments reporting lesion lengths associated with inoculation with the following fungi (in order of decreasing virulence): *N. parvum*, *E. lata*, and *D. seriata* [37]. *Fomitiporia polymorpha* WFB1 was originally isolated from decayed wood, which appeared to be a white rot-type of decay (S1 Fig), and later isolate WFB1 was demonstrated to be pathogenic [26]. Because of variable growth rates of the four species, there were different incubation periods to reach similar colony diameter at the edge of a 100-mm Petri plate, at 25˚C: 5 days for *N. parvum* and *D. seriata*, 10 days for *E. lata*, and 21 days for *F. polymorpha*. When inoculated to media or wood blocks, agar plugs were taken from the colony margin of such ‘PDA plates’.

### Wood block-decay assay

**Fig 1:**
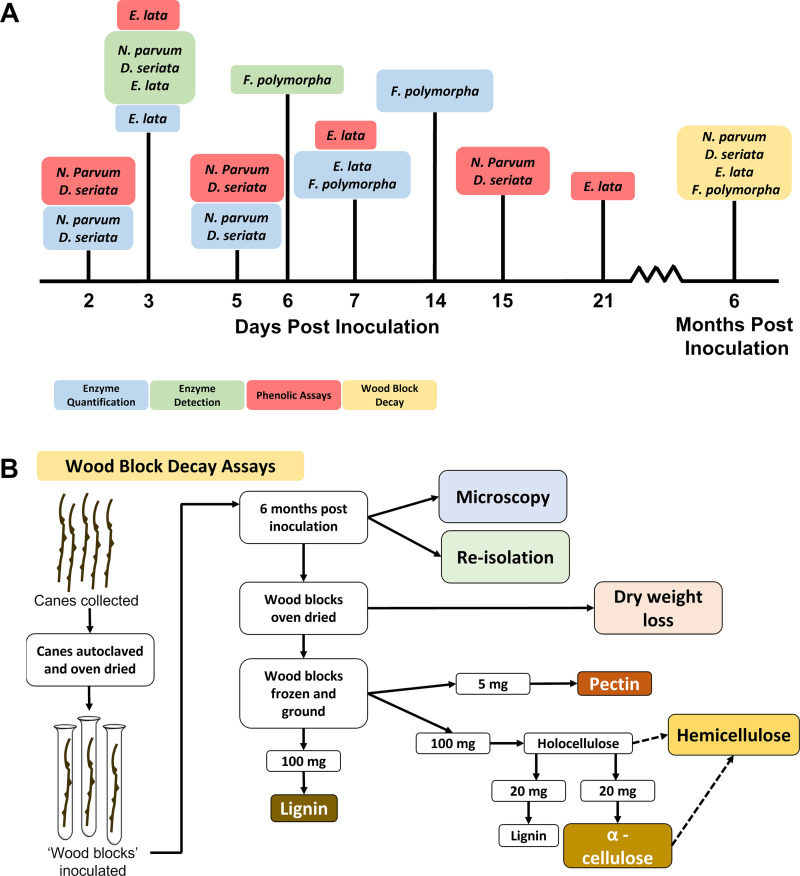
Assays to characterize wood-decay abilities. (A) Incubation periods of *D. seriata*, *E. lata*, *F. polymorpha*, and *N. parvum*, during *in vitro* assays (enzyme quantification, enzyme detection, phenolic assays, and wood block decay). (B) Steps of wood block-decay assays, showing when samples were collected for microscopy, re-isolation, and quantitation of dry-weight loss and percentages (dry weight) of cell-wall components (lignin, pectin, α-cellulose, and hemicellulose) remaining.

Wood blocks were inter-nodal segments of 2-year-old, woody canes of *Vitis vinifera* ‘Merlot’ (approx. 4-cm in length x 8-mm diam.), collected from a vineyard (Armstrong Field Station, Department of Plant Pathology, University of California, Davis; Wood block-decay assays, Fig 1A). Canes were debarked and autoclaved twice for 90 min, with 48 h between the first and second autoclaving. Canes were then oven-dried at 50°C for 72 h, weighed, and re-hydrated for 36 h in sterile distilled water. The resulting wood blocks were set inside individual glass tubes under aseptic conditions and then inoculated with a 6-mm agar plug of a PDA culture (30 wood blocks per each of *N. parvum*, *D. seriata*, *E. lata*, and *F. polymorpha*) or 30 mock-inoculated wood blocks (control) with sterile PDA. Tubes were capped with cotton, to maintain moisture and sterility, and then incubated at 25°C for 6 months.

At 6 months post-inoculation, four of the 30 wood blocks per fungus were collected for re-isolation of the inoculated fungus [37], to confirm viability of the fungi during the assay (Fig 1B). Six of the 30 samples per inoculation treatment were collected for microscopy, as described below (see ‘Microscopic fungal colonization of wood blocks’). The remaining 20 samples were oven dried for 72 h at 50°C and weighed to measure dry weight loss [percent dry mass = weight initial – (weight final/weight initial) ×100] (Fig 1B). After weighing, the oven-dried wood blocks were frozen in liquid nitrogen and stored at −80°C. Frozen wood blocks were ground to a fine powder in liquid nitrogen using a tissue lyser (Retsch, Newtown, PA, USA) with steel jars and 1-cm diameter steel balls. Each sample was subsequently divided into three aliquots (two 100-mg and one 5-mg amounts). Soluble phenolic compounds were removed from the three wood aliquots by treating with 500 μl methanol overnight at 4°C, removal of methanol, and re-extraction of the pellet with an additional 500 μl methanol overnight at 4°C [33]. Methanol supernatants were discarded, and the pellets were washed with 1 ml of sterile water, then vacuum dried and weighed.

#### Lignin extraction.

A thioglycolic acid-based method was used to extract lignin [38]. Briefly, one 100-mg pellet (as described above) was suspended in 400 μl 1 N NaOH (Millipore-Sigma) and incubated at 40°C with gentle shaking for 21 h. The solution was acidified with 400 μl of 1.5 M formic acid (Fisher Scientific) followed by the addition of 200 μl methanol, and the supernatant was discarded. Following a sterile water wash, the pellet was treated with 800 μl of 2 N HCl (Fisher Scientific) and 300 μl of thioglycolic acid (Fisher Scientific), and incubated for 4 h at 86°C. The supernatant was discarded. The pellet was washed twice in sterile water, and then was re suspended in 1 ml of 0.5 M NaOH on a shaker (500 rpm) for 18 h. The NaOH supernatant was transferred to 2-ml centrifuge tubes, with the pellet again suspended in 0.5 ml of 0.5 M NaOH on a shaker for 18 h. The supernatants then were combined, and 300 μl of concentrated HCl were added with the mixture incubated at room temperature for 4 h. The supernatant then was discarded, and the pellet containing bound lignin was re suspended in 1 ml of 0.5 M NaOH. For the analysis, 2 μl of this solution were mixed with 98 μl of 0.5 M NaOH and read at 280 nm on the Biotek Epoch microplate reader (Agilent Technologies). Absorbance values were recorded and compared with a standard curve of lignin (Millipore-Sigma) suspended in 0.5 M NaOH at concentrations of 0.01, 0.10, 0.25, 0.5, and 1 mg/ml to calculate the total weight of lignin in each sample. Values were then converted to percentage of remaining weight.

#### Hemicellulose extraction.

The second 100-mg pellet was used to extract holocellulose and α-cellulose, which allows for the calculation of hemicellulose [39]. The pellet was washed with 1 ml of 100% acetone, dried under a vacuum, and weighed. Tubes were placed at 70°C, while 1.5 ml of 0.2% sodium acetate (pH 3.2) and 200 μl 20% sodium chlorite were added. The solution was mixed with inversion to fully wet the pellet. After 45 min, the tubes were moved directly to an ice-cold water bath for 5 min, and the supernatant was removed. The pellet was subsequently washed with 1 ml of ice-cold 1% acetic acid three times, ice-cold 100% acetone twice, and then dried under vacuum to determine the holocellulose weight. The resulting holocellulose fraction was divided into two 20-mg aliquots. Lignin was extracted from the first aliquot (using the protocol described above [39], but modified to utilize only one-fourth the reagent volumes), and α-cellulose was extracted from the other aliquot.

#### Cellulose extraction.

The 20-mg pellet for α-cellulose extraction was washed with 1 ml of 100% acetone and transferred to a 20°C water bath. Two hundred μl of 17.5% NaOH were added and the samples were mixed gently by inversion and incubated in the 20°C water bath for 5 min. Four hundred μl of 17.5% NaOH were added to the samples. They were mixed gently by inversion and incubated for 40 min in the 20°C water bath. Four hundred μl of sterile water were added to the samples and the supernatants were removed. The pellets were washed with 1 ml of sterile water three times and dried under vacuum. The resulting samples were weighed to obtain α-cellulose values. The milligram weight of lignin extracted from holocellulose and milligram weight of α-cellulose extracted were subtracted from holocellulose to obtain weight of hemicellulose in the wood blocks. Cellulose and hemicellulose values were converted to percentages of weight remaining.

#### Pectin extraction.

The 5-mg pellets were used to extract pectin and were measured via a colorimetric assay [40,41]. Briefly, the pellet was placed in a glass test tube with 2 ml of chilled concentrate sulfuric acid, with gentle stirring in an ice-water bath. Then 500 μl of sterile water were added dropwise, samples were stirred for an additional 5–10 min, and another 500 μl of sterile water were added until dissolution was complete. The volume was adjusted to 10 ml with sterile water. From this solution, 600 μl were placed into a clean glass test tube and 3.6 ml of chilled 12.5 mM sodium tetraborate in concentrate sulfuric acid were added, and gently stirred. The samples were placed in boiling water for 5 min and the tubes cooled in a room temperature water bath. Then 120 μl of this solution were placed into a flat-bottom microplate and 2 μl of 0.15% m-phenylphenol in 0.5% NaOH were added to each of the sample wells. Each sample also had a blank well, which contained only 2 μl 0.5% NaOH. The plate was measured promptly at 520 nm. Absorbance values from blank wells were subtracted from sample wells. Pectin weight was calculated based on a standard curve of polygalacturonic acid of 1, 5, 10, 25, and 50 μg/ml. Total weight was converted to percentage of weight remaining. Lastly, an estimate of the remainder of the wood blocks (“All Other” compounds) was made by subtracting the percentages that cellulose, hemicellulose, lignin, and pectin comprised of the wood from 100%.

All statistical analyses were performed using SPSS version 24.0 (IBM). Normality, homogeneity of variance, and outliers were evaluated using Anderson-Darling Test, Levene’s Test, and Outlier Test, respectively. Separate ANOVAs were used to determine the effect of inoculation treatment (non-inoculated, *N. parvum*, *D. seriata*, *E. lata*) on percent dry weight loss and percent dry weight remaining of cell-wall components (lignin, pectin, cellulose, and hemicellulose). For significant effects (*F* values with *P* < 0.05), means were compared using the Tukey-Kramer method (∝ = 0.05), with *P*-values and 95% confidence limits for mean differences adjusted for multiple comparisons.

#### Microscopic fungal colonization of wood blocks.

After 6 months, six wood blocks inoculated with each fungus and six non-inoculated controls were fixed in 2% glutaraldehyde/2% formaldehyde in Phosphate Buffer Saline (pH 7.2) (PBS) to stop fungal growth (Fig 1B). Samples were soaked in the fixative solution for 48 h, then rinsed with PBS three time over 72 h. Samples were then mounted for sectioning using a sledge microtome (American Optical Company, Buffalo, NY, USA) and a tungsten carbide knife (Delaware Diamond Knives, Wilmington, DE, USA). Transverse and longitudinal sections were made, approx. 30 µm in thickness. To visualize fungal hyphae and wood tissues, sections were stained with Pianze IIIb [42] and observed with epifluorescence under green excitation (Y3 filter cube) on a compound microscope (Leica DM5000B, Leica Microsystems, Heidelberg, Germany) from 200 to 400X magnification.

### Enzyme-detection assay

The ability of each fungus to grow and to produce extracellular enzymes, when grown on solid medium amended with individual wood components, was assessed (Fig 1A). Minimal medium was Eriksson and Pettersson medium [43], solidified with 2% bacto-agar (Fisher Scientific, Pittsburg, PA, USA). Amendments were 2% (*w/v*) of the following compounds [from Millipore-Sigma (St. Louis, MO, USA), unless otherwise indicated]: starch, pectin (adjusted to pH 5 and pH 7), lignin, carboxymethylcellulose (CMC, ‘cellulose’), birch xylan (‘hemicellulose’; Megazyme; Wicklow, Ireland, UK), tannic acid, gallic acid, and manganese sulfate. Tannic and gallic acids were added after autoclaving, to prevent hydrolyzation of the agar. Amended media were inoculated with a 4-mm PDA plug from an actively growing culture as described above (*n* = 6 per medium × fungus combination). Amended media inoculated with *E. lata*, *D. seriata*, and *N. parvum* were incubated for 3 days, and those inoculated with *F. polymorpha* were incubated for 6 days, all at 25°C in the dark. Plates containing manganese sulfate were incubated for 3–6 days, as described above, at 25°C in 12 hours light/12 hours dark. The different incubation times, given different growth rates among the four fungi, were sufficient for at least 3-mm of growth from the agar plug or visible discoloration of the plate 3-mm from the plug. Colony diameter was measured bidirectionally on amended and unamended plates before processing the medium for detecting enzyme activity. This experiment was duplicated, two weeks after the first experiment was started.

We examined medium amended with starch, pectin, lignin, cellulose, hemicellulose, tannic acid, gallic acid, or manganese sulfate for activity of the following enzymes, respectively: amylase, pectinase, pectin lyase, lignin oxidation, cellulase, hemicellulase, laccase, manganese peroxidase, and polyphenol oxidase [44]. Amylase was detected as a clear ‘halo’ (i.e., removal of the black color), after adding Lugol’s iodine solution (Fisher Scientific) for 2 min to the surface of the starch-amended medium (S1 Fig). Pectinases and pectin lyases were detected as a clear halo, after adding 1% CTAB to the surface of the pectin-amended medium. Lignin oxidation was detected as a dark-brown color change in lignin-amended medium. Cellulase activity was detected as a yellow color change on a red background, after flooding cellulose-amended plates with 1 mg/ml Congo Red (Millipore-Sigma) for 15 minutes and destaining with 1 M NaCl. Hemicellulase activity was detected as a purple halo on a red background by flooding hemicellulose-amended plates with 1 mg/ml Congo Red for 15 minutes and destaining with 1 M NaCl. Laccase and polyphenol-oxidase activities were detected by a brown color change in media amended with tannic and gallic acids, respectively. Manganese-peroxidase activity was detected as dark crystals forming on manganese sulfate-amended medium [45]. Color-change diameter was measured bidirectionally on amended and unamended plates.

All statistical analyses were performed using SPSS version 24.0 (IBM, Armonk, NY, USA). Normality, homogeneity of variance, and outliers were evaluated using Anderson-Darling Test, Levene’s Test, and Outlier Test, respectively. T-tests were used to compare mean colony diameters on amended media to those of unamended media of each fungus, to determine whether each cell-wall component (starch, pectin, lignin, cellulose, hemicellulose, tannic acid, gallic acid, or manganese sulfate) significantly affected fungal growth. No color change (i.e., no enzyme activity) was assessed or observed in the unamended media, as this was a qualitative assay for enzyme activity.

### Enzyme-quantification assay

The enzymatic activities of lignin peroxidase, peroxidase, polyphenol oxidase, cellulase, and hemicellulase enzymes (Fig 1A) were quantified [46,47]. Each medium was inoculated with a 4-mm PDA plug from an actively growing culture, as described above. Fungi were incubated in liquid minimal medium, either amended with 20 mg/ml of ground wood of *V. vinifera* ‘Merlot’ or unamended (control), at 25°C, 150 rpm. Enzyme activity was quantified at 2, 2, 3, or 7 days (‘early’) and 5, 5, 7, or 14 days (‘mid’), for *D. seriata, E. lata, N. parvum*, or *F. polymorpha*, respectively (Fig 1A). Each fungus was grown in five replicate tubes per timepoint, and the experiment was repeated twice (*n* = 10 per culture medium, per timepoint, per fungus). At the time of collection, culture media were filtered through the following three filters: (1) cheese cloth, (2) Whatman GF/A filter paper (70-mm diameter; 1.6-μm pore), and (3) Whatman GF/F filter paper (70-mm diameter; 0.7-μm pore).

To quantify lignin-peroxidase activity, 50 μl of culture supernatant were added to 100 μl of 100 mM sodium tartrate (pH 4.5), 30 μl ultrapure water, and 10 μl of 640 μM Azure B. Then 1 mM H_2_O_2_ was added to each well, and the decrease in absorbance was read promptly at 650 nm with a 30 min kinetic read (5 min reads) on a Biotek Epoch microplate reader (Agilent Technologies, Santa Clara, CA, USA) [47]. To quantify peroxidase activity, 50 μl of culture supernatant were added to 150 μl of 0.25% guaiacol in 0.1 M sodium phosphate (dibasic, anhydrous) buffer pH 6, containing 0.3% H_2_O_2_ in a microplate. Plates contained three water blanks, a horseradish-peroxidase standard curve with 0.2, 1, 1.3, 2, and 5-mg/ml solutions (Millipore-Sigma), and samples, all of which were incubated for 5 min at room temp, and then read at 470 nm [46]. To quantify polyphenol-oxidase activity, 50 μl of culture supernatant were added to 150 μl of 3-mM caffeic acid (Millipore-Sigma) in 0.05 M sodium phosphate (dibasic, anhydrous) buffer pH 8 in a microplate. Plates contained three water blanks, a tyrosinase (from mushroom) standard curve with 10, 40, 100, 250, and 500-μg/ml solutions (Millipore-Sigma), and samples, all of which were incubated for 5 min at room temp, and then read at 470 nm [46].

Cellulase (endo-1,4-β-glucanase) and hemicellulase (endo-1,4-β-xylanase) assays were adapted from Megazyme protocols [47]. Cellulase and hemicellulase activities were determined by measuring the removal of Remazol Brilliant Blue from carboxymethylcellulose for cellulase (Megazyme S-ACMC) and from birchwood xylan, respectively (Megazyme S-AXBP). Preparations of substrates and precipitation buffers followed the manufacturer’s protocols. For both protocols, 70 μl of culture supernatant were added to 70 μl of substrate solution in a microplate. Plates were incubated at 40°C in a Shake ‘N Bake Incubator (Boekel Scientific, Feasterville, PA, USA), at 10 strokes per minute, for 2 h for cellulase and 4 h for hemicellulase. After incubating, 200 μl of precipitation buffer were transferred to a new microplate and 80 μl of the incubation solution were added, followed by vigorous pipetting. Both cellulase and hemicellulase plates were incubated for an additional 10 min at room temperature in the corresponding precipitation buffer. Plates were then centrifuged at 1,000 x g for 15 min, and 150 μl of the supernatant were transferred to a new plate, which was then read at 590 nm on a Biotek Epoch microplate reader (Agilent Technologies). Each plate contained a calibration curve of Remazol Brilliant Blue in the sodium acetate buffer provided for each enzyme assay. The enzyme activities were calculated from the concentration of released Remazol Brilliant Blue.

All statistical analyses were performed using SPSS version 24.0 (IBM). Normality, homogeneity of variance, and outliers were evaluated using Anderson-Darling Test, Levene’s Test, and Outlier Test, respectively. ANOVAs were used to determine the effects of fungus (*N. parvum*, *D. seriata*, *E. lata*, *F. polymorpha*), time point (early, mid), and their interaction on enzyme activity of lignin peroxidase, peroxidase, polyphenol oxidase, cellulase, and hemicellulase. For significant effects (*F* values with *P* < 0.05), means were compared using the Tukey-Kramer method (∝ = 0.05), with *P*-values and 95% confidence limits for mean differences adjusted for multiple comparisons.

### Phenolic assays

The ability of *N. parvum*, *D. seriata*, and *E. lata* to grow on and metabolize phenolic compounds as their sole carbon source was assessed in liquid minimal medium (Fig 1A). Basidiomycete *F. polymorpha* was excluded from this assay, due to its inability to grow on gallic acid (no growth was observed after 30 d), in the enzyme-detection assay. Representatives from four classes of phenolic compounds were added as amendments, at concentrations found in healthy grape wood [33,48]: hydrolysable tannins were represented by gallic acid (2 mg/ml; Millipore-Sigma); stilbenes were represented by piceid (4 μg/ml; Millipore-Sigma); flavonols were represented by rutin (50 μg/ml; Millipore-Sigma); and proanthocyanidins/ catechins were represented by epicatechin (2 mg/ml; Millipore-Sigma). Controls included unamended medium inoculated with a 4-mm agar plug of *E. lata*, *D. seriata*, or *N. parvum*, and amended media ‘mock-inoculated’ with a sterile PDA plug. Incubation periods were as follows: *D. seriata* and *N. parvum* supernatant for 2 days (‘early’), 5 days (‘mid’), and 15 days (‘late’), and *E. lata* supernatant for 3 days (‘early’), 7 days (‘mid’), and 21 days (‘late’) (Fig 1A). Each medium × inoculation treatment × timepoint had six replicates, in two experiments (*n* = 60 samples per inoculation treatment per experiment; 720 samples total). This experiment was duplicated, two weeks after the first experiment was started. Four isolates per fungus were evaluated for intraspecies differences, but differences were negligible within a species, and so we present the results for the one isolate per species examined for the wood block-decay and enzyme assays.

At the end of each incubation period, the culture medium for all samples was filtered through Whatman GF/A filter paper (Millipore-Sigma), fungal biomass was weighed, and the culture filtrate was collected to quantify compound concentrations and possible derivatives. Filtrates were analyzed using a Shimadzu (Columbia, MD, USA) high-performance liquid chromatography (HPLC) system, that was based on Shimadzu LC-20AD pumps, equipped with an Supelco Ascentis (Millipore-Sigma, St. Louis, MO) C18 reverse-phase column kept at 50°C, and detection made by observing the 280 nm wavelength with a Shimadzu SPD-20 photodiode array detector. For each sample, 50 μl were injected and a binary gradient used to proceed from 95% solvent A (water with 2% v/v glacial acetic acid; Fisher Scientific) to 100% solvent B [methanol (Fisher Scientific) with 2% v/v glacial acetic acid] for a total retime of 40 min [46]. Compounds were identified and quantified by performing external standard runs of the compounds.

All statistical analyses were performed using SPSS version 24.0 (IBM). Normality, homogeneity of variance, and outliers were evaluated using Anderson-Darling Test, Levene’s Test, and Outlier Test, respectively. ANOVAs were used to determine the effect of fungus (*N. parvum*, *D. seriata*, *E. lata*), time point (early, mid), and their interaction on compound concentration (gallic acid, rutin, piceid, epicatechin). T-tests were used to compare fungal biomass (fresh weight) in amended media to those of unamended media of each fungus, to determine whether each phenolic compound (epicatechin, gallic acid, piceid, or rutin) significantly affected fungal growth.

## Results

### Wood block-decay assay

All four fungi colonized the wood blocks and were re-isolated without contaminating fungi. The percent dry weight loss of all inoculated wood blocks after six months was statistically greater than that of the non-inoculated controls (*P* = 0.01, Table 1). Percent dry weight loss of wood blocks inoculated with *D. seriata* was greatest, *F. polymorpha* was the least, and *E. lata* and *N. parvum* were intermediate. Inoculation treatment had a significant effect on percent dry weight remaining of lignin, pectin, cellulose, and hemicellulose (*P* < 0.05 for all four cell-wall components). Blocks inoculated with *D. seriata* had significantly higher percentage of remaining lignin, compared to all other treatments, including the control. It is important to note that the lignin extract from *D. seriata*-inoculated wood blocks was visibly darkest. Blocks inoculated with *N. parvum*, *F. polymorpha*, or *D. seriata* had significantly lower percentages of remaining pectin, compared to the control. Blocks inoculated with *E. lata* or *F. polymorpha* had significantly higher percentages of remaining cellulose, compared to all other treatments, including the control. Blocks inoculated with *F. polymorpha* had significantly lower percentage of remaining hemicellulose than all other treatments (25% that of the control) and blocks inoculated with *N. parvum* had significantly higher percentage of remaining hemicellulose.

**Table 1:** Percent dry weight loss and percent dry weight remaining of cell-wall components (lignin, pectin, cellulose, and hemicellulose) at 6 months post-inoculation of *Vitis vinifera* ‘Merlot’ wood blocks inoculated with *D. seriata*, *N. parvum*, *E. lata*, and *F. polymorpha*, versus a non-inoculated control. Values represent the means of 20 blocks per inoculation treatment (95% confidence limits are shown in parentheses). Means with different letters within a column are significantly different (Tukey’s test, *P* ≤ 0.05).

Inoculation	% Dry weight loss	Cell-wall components (% Dry weight remaining)			
		Lignin	Pectin	Cellulose	Hemicellulose
Control	1.9 a (1.3, 2.5)	20.9 a (17.7, 24.2)	7.5 a (6.3, 8.7)	31.0 a (29.3, 32.0)	12.2 b (7.5, 18.0)
*D. seriata*	13.4 d (12.8, 14.3)	53.3 b (50.2, 56.5)	5.1 bc (3.8, 6.3)	31.1 a (29.3, 33.0)	15.8 b (10.4, 22.3)
*N. parvum*	10.4 c (9.8, 11.1)	27.2 a (24.0, 30.3)	2.7 c (1.5, 3.9)	27.7 a (26.1, 29.4)	45.8 c (36.0, 56.8)
*E. lata*	11.2 c (10.8, 12.2)	24.9 a (21.8, 28.1)	5.9 ab (4.7, 7.2)	35.4 b (33.3, 37.6)	9.3 ab (5.3, 14.5)
*F. polymorpha*	5.8 b (5.2, 6.4)	24.2 a (21.1, 27.4)	3.8 bc (2.6, 5.0)	34.1 b (33.1, 37.2)	3.6 a (1.3, 7.0)

### Microscopic fungal colonization of wood blocks

**Fig 2:**
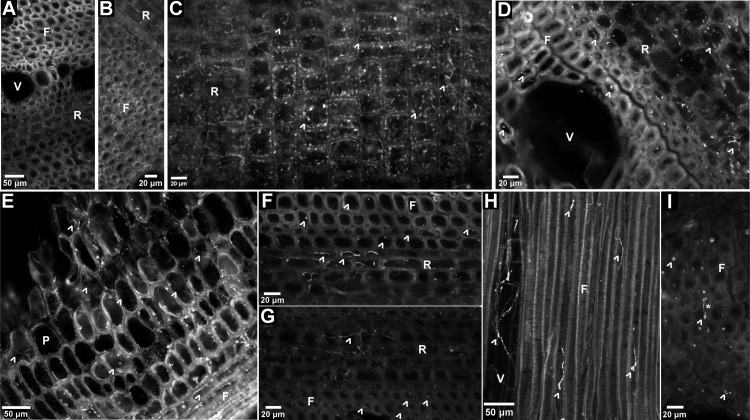
Localization of hyphae in wood blocks, at 6 months post-inoculation. (A) Transverse section of non-inoculated wood block, with hyphae absent from xylem fibers (F), rays (R), and vessels (V). (B) Transverse section of non-inoculated wood block, with hyphae absent from xylem rays (R) and fibers. (C) Longitudinal section of ray parenchyma extensively colonized by hyphae (^) of *D. seriata* and parenchyma cell walls visibly damaged/deteriorated. (D) Transverse section with hyphae of *D. seriata* localized in fibers and rays. (E) Longitudinal section of pith parenchyma and bordering fibers colonized by hyphae of *N. parvum*. (F) Transverse section of ray and fiber lumens colonized by hyphae of *N. parvum*. (G) Transverse section of fibers and rays colonized by hyphae of *E. lata*. (H) Longitudinal section of fibers and a vessel colonized by hyphae of *F. polymorpha*. (I) Transverse section of fibers colonized by hyphae of *F. polymorpha*, which span cell walls of two adjacent fibers (*).

Compared to non-inocuated wood blocks (Fig 2A,B), wood blocks inoculated with *D. seriata* had denser hyphae and more extensive damage to the wood cell walls. *Diplodia seriata* colonized the xylem ray parenchyma predominantly (Fig 2C), but also colonized fibers and vessels (Fig 2D). Pith parenchyma and bordering fibers were colonized by *N. parvum* extensively (Fig 2E), compared to relatively sparse colonization of the xylem fibers and rays (Fig 2F). *Eutypa lata* colonized the xylem ray parenchyma (predominantly), fibers, and vessels (Fig 2G), although the hyphae were more sparse in all three cell types, compared to that of *D. seriata* (Figs 2C,D). *Fomitiporia polymorpha* colonized only xylem fibers and vessels (Fig 2H), and was the only fungus with hyphae spanning adjacent fibers (i.e., colonizing their cell walls and the middle lamella between them; Fig 2I).

### Enzyme-detection assay

**Fig 3:**
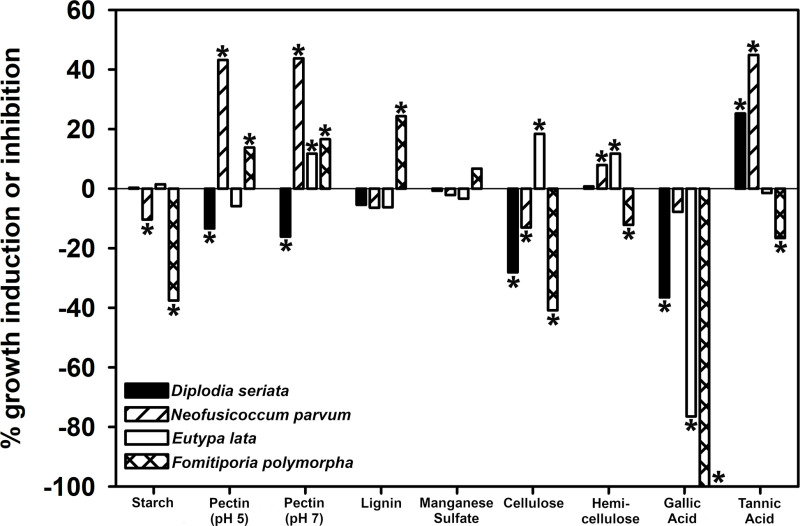
Percentage of fungal-growth induction (> 0) or inhibition (< 0) by nine wood components. Timepoints are 3 days (‘early’) for *D. seriata, E. lata,* and *N. parvum*, and 6 days (‘early’) for *F. polymorpha*. Fungi were grown on solid minimal medium amended with each wood component, compared to an unamended control (Enzyme-detection assay, Fig 1A). Each bar is the difference in mean colony diameters of 12 amended plates versus 12 unamended plates per fungus, pooled across two replicate experiments. Bars with an asterisk represent significant larger mean colony diameters (> 0) or smaller mean colony diameters (< 0) on amended versus unamended media (T test, ****P**** ≤ 0.05). Bars with no asterisk represent no statistically significant effect of the wood component on fungal growth.

No wood component significantly induced growth of all four fungi, compared to their growth in unamended media (Fig 3). Growth of all four fungi was either unaffected (i.e., no statistically significant difference in mean colony diameters on amended media versus unamended media; *N. parvum*) or significantly inhibited (i.e., mean colony diameters on amended media were significantly lower than those of unamended media; *D. seriata, E. lata, F. polymorpha*) by gallic acid. Growth of all four fungi was either unaffected (*D. seriata*, *E. lata*) or significantly inhibited (*N. parvum*, *F. polymorpha*) by starch. Growth of all four fungi was unaffected by manganese sulfate. Growth of *D. seriata* was either unaffected or significantly inhibited by all compounds, except tannic acid, which significantly induced *D. seriata* growth. Growth of *N. parvum* was significantly induced by pectin (pH 5 and 7), hemicellulose, and tannic acid. Growth of *E. lata* was significantly induced by pectin (pH 7 only), cellulose and hemicellulose. Growth of *F. polymorpha* was significantly induced by pectin (pH 5 and 7) and lignin.

**Fig 4:**
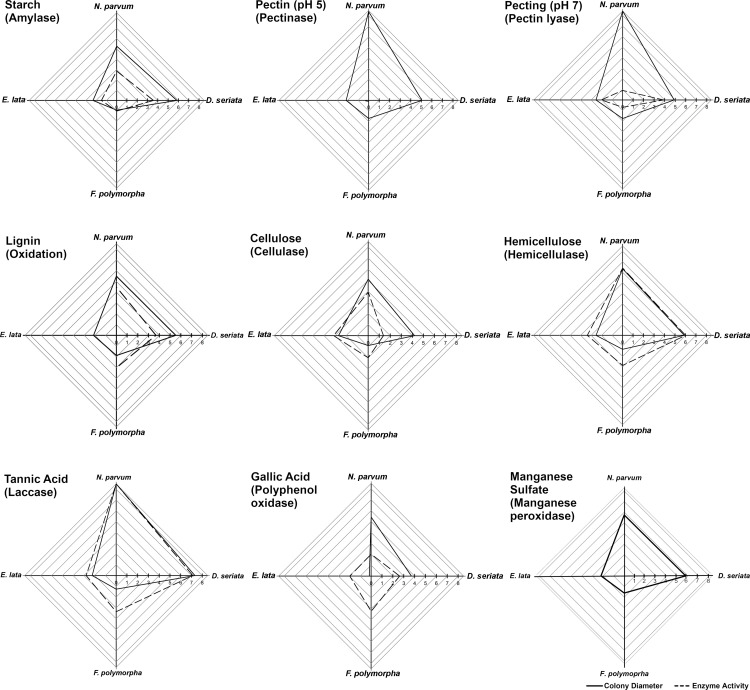
Mean colony diameter (solid line) and detection of enzyme activity (i.e., mean color-change diameter, dashed line). Timepoints are 3 days (‘early’) for *D. seriata, E. lata,* and *N. parvum*, and 6 days (‘early’) for *F. polymorpha*. Fungi were grown on solid minimal medium amended with each wood component (Enzyme-detection assay, Fig 1A). No enzyme activity was detected in unamended controls. Values on the axes represent the mean-colony and color-change diameters of 12 plates per fungus, pooled across two replicate experiments (maximum value is 8.5 cm).

Activities of amylase, cellulase, hemicellulase, laccase, pectin lyase (pH 7), and polyphenol oxidase were detected among all four fungi (Fig 4). There was no color change for any of the fungi on plates amended with pectin at pH 5 (i.e., no pectinase activity) or plates amended with manganese sulfate (i.e., no manganese-peroxidase activity; S2 Fig). The only fungus that did not oxidize lignin was *E. lata*.

### Enzyme-quantification assay

**Fig 5:**
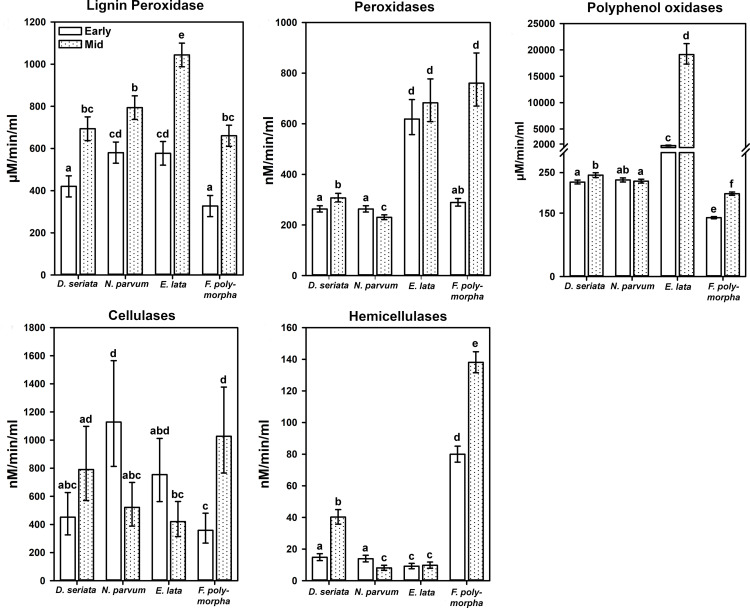
Quantification of enzyme activity. Timepoints are 2, 2, 3, or 7 days (‘early’) and 5, 5, 7, or 14 days (‘mid’), for *D. seriata, E. lata, N. parvum*, or *F. polymorpha*, respectively. Fungi were grown in liquid minimal medium amended with grapevine wood powder, compared to an unamended control (Enzyme-quantification assay, Fig 1A). Bars represent the mean of 20 tubes of amended media per fungus, pooled across two replicate experiments (no enzyme activity was detected in unamended controls). Error bars are 95% confidence limits. Means with different letters within a panel are significantly different (Tukey’s test, ****P**** ≤ 0.05).

With wood powder as substrate in liquid minimal medium, as opposed to specific wood components in solid minimal medium for the enzyme-detection assays, enzymatic activity shared in common among all four fungi included cellulases, hemicellulases, lignin peroxidases, peroxidases, and polyphenol oxidases (Fig 5). With twice the incubation period as in the enzyme-detection assays, *E. lata* had the highest activity among all four fungi of lignin peroxidases and especially polyphenol oxidases. A consistent finding for both the enzyme-detection assay (Fig 4) and enzyme-quantification assay was the high polyphenol-oxidase activity of *E. lata*, which was 100x higher than that of all other fungi in the latter assay (Fig 5). *Neofusicoccum parvum* and *E. lata* had the highest cellulase activity at the ‘early’ timepoint, whereas similar levels of cellulase activity were not reached by *D. seriata* or *F. polymorpha* until the ‘mid’ timepoint. *Fomitiporia polymorpha* had the highest hemicellulose activity in the enzyme-detection assay (Fig 4) and the enzyme-quantification assay. In the latter assay, this activity was at least 4x higher than that of the other fungi (Fig 5).

### Phenolic assays

**Fig 6:**
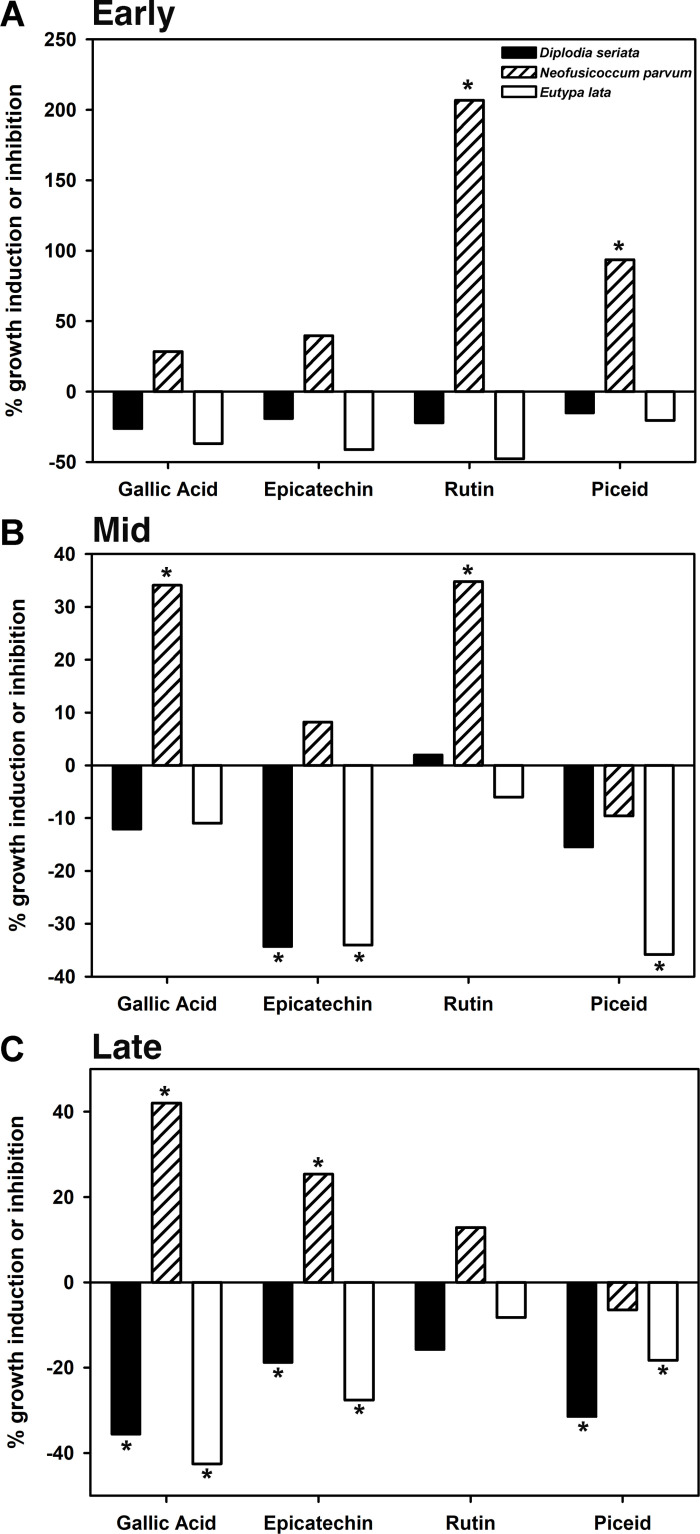
Percentage of fungal-growth induction (> 0) or inhibition (< 0) by four phenolic compounds. Timepoints are: (A) 2 or 3 days (‘early’), (B) 5 or 7 days (‘mid’), and (C) 15 or 21 days (‘late’) for *D. seriata* and *N. parvum*, or *E. lata*, respectively. Fungi were grown in liquid minimal medium amended with four phenolic compounds, compared to an unamended control (Phenolic assays, Fig 1A). Each bar is the difference in mean fungal biomass (fresh weight) of 12 amended tubes versus 12 unamended tubes per fungus, pooled across two replicate experiments. Bars with an asterisk represent significant larger mean fungal biomass (> 0) or smaller mean fungal biomass (< 0) on amended versus unamended media (T test, ****P**** ≤ 0.05). Bars with no asterisk represent no statistically significant effect of the phenolic compound on fungal growth.

The three Ascomycetes (*D. seriata, E. lata, N. parvum*) grew in medium amended with phenolic compounds and exhibited polyphenol oxidase activity (Figs 4 and 5), and thus were further investigated for their *in vitro* ability to tolerate the phenolic compounds epicatechin, gallic acid, piceid, and rutin, at concentrations found in healthy grapevine wood. Because *F. polymorpha* did not grow on gallic acid (Fig 4), and had significantly lower polyphenol-oxidase activity than the three Ascomycetes (Fig 5), *F. polymorpha* was excluded from the phenolic assays. Growth of *N. parvum* was induced by epicatechin, gallic acid, piceid, and rutin, with inhibition of growth only by piceid at the mid and late timepoints (Fig 6). This was unique among the three Ascomycetes. In contrast, *D. seriata* growth was induced only by rutin and only at the mid timepoint, and *E. lata* growth was consistently inhibited at all time points by all four compounds.

**Fig 7:**
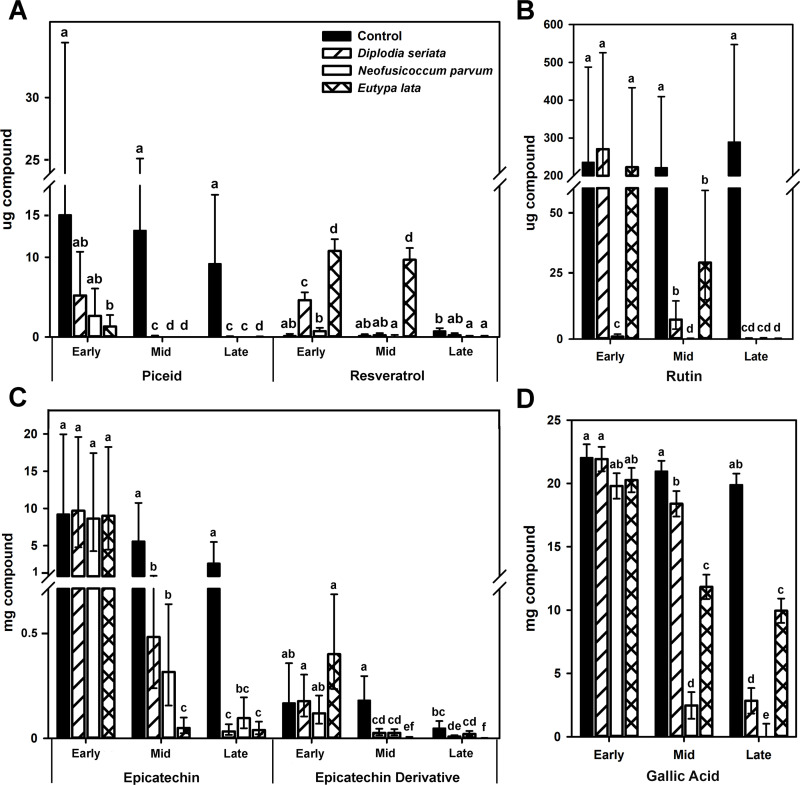
Phenolic compounds remaining in liquid minimal medium, after incubation. Phenolic compounds are: (A) Piceid and the developed resveratrol derivative, (B) Rutin, (C) Epicatechin and a developed epicatechin derivative, and (D) Gallic acid. Timepoints are: 2 or 3 days (‘early’), 5 or 7 days (‘mid’), and 15 or 21 days (‘late’) for *D. seriata* and *N. parvum*, or *E. lata*, respectively. Fungi were grown in liquid minimal medium amended with four phenolic compounds, compared to non-inoculated control tubes with each phenolic compound (Phenolic assays, Fig 1A). Bars represent the mean of 12 tubes per fungus. Error bars are 95% confidence limits. Means with different letters within a panel are significantly different (Tukey’s test, ****P**** ≤ 0.05). Resveratrol was derived from elimination (by the fungi) of glucose from piceid.

Eventually, all three Ascomycetes significantly reduced the concentrations of or completely eliminated from the media epicatechin, piceid, and rutin, compared to those of the non-inoculated controls (Fig 7). *Eutypa lata* reduced piceid to the lowest concentrations of all three Ascomycetes at the early timepoint, compared to that of the non-inoculated control, but only at the late timepoint did *E. lata* significantly reduce the resveratrol that had accumulated from reduction of piceid (Fig 7A). *Neofusicoccum parvum* almost eliminated rutin from the medium at the ‘early’ timepoint, but significant rutin depletion did not happen until the ‘mid’ timepoint for the *D. seriata* or *E. lata*-inoculated tubes (Fig 7B). *Eutypa lata* almost eliminated epicatechin (and the resulting epicatechin derivative) from the medium at the ‘mid’ timepoint. The *D. seriata* and *N. parvum*-inoculated tubes exhibited the same reduction in epicatechin levels as that of *E. lata*, but only at the 'late' timepoint (Fig 7C). *Eutypa lata* was the least efficient at lowering the concentration of gallic acid and was only able to remove 50% of the available gallic acid (compared to the non-inoculated control) by the ‘late’ timepoint (Fig 7D).

## Discussion

In comparing the wood-colonization capabilities among species that cause the grapevine trunk diseases Botryosphaeria dieback (*D. seriata*, *N. parvum*), Esca (*F. polymorpha*), and Eutypa dieback (*E. lata*), we set out to clarify the types of wood decay they cause. We identified novel phenotypic characteristics of *F. polymorpha*, an Esca pathogen unique to North America [49], from examination of an isolate previously identified from vines with leaf and wood symptoms of Esca, and later confirmed to be pathogenic in the greenhouse [26]. This *F. polymorpha* isolate had characteristics of both brown-rot and white-rot fungi. From past studies of Basidiomycetes that are brown-rot fungi, characteristics of brown-rotted wood (in comparison to non-inoculated controls) include low percent wood dry-weight loss and significant degradation of hemicellulose and pectin, but little-to-no degradation of lignin [50,51]. In wood-block assays with Basidiomycetes that are white-rot fungi, the inoculated wood blocks are characterized by high percent wood dry-weight loss, and degradation of cellulose, hemicellulose, pectin, and lignin [50,51]. Our findings from the grape wood blocks inoculated with *F. polymorpha*, namely significant loss of hemicellulose and pectin, but not of lignin (compared to the non-inoculated controls), suggest that *F. polymorpha* shares this characteristic with brown-rot fungi.

From the enzyme-detection and quantification assays with *F. polymorpha*, activities of pectin lyase, cellulase, and especially hemicellulase, but no activity of manganese peroxidase, are consistent with those of other brown-rot fungi [50,51]. Nonetheless, *F. polymorpha* had *in vitro* activities of enzymes involved with lignin degradation (laccase, lignin peroxidase, peroxidase, and polyphenol oxidase). In lignin-amended medium, there was strong oxidation of lignin and induction of *F. polymorpha* growth, all of which are characteristics of a white-rot fungus [50,51]. Furthermore, comparative analyses of the genomes of *F. polymorpha*, *F. mediterranea*, and other white-rot fungi (e.g., *Trametes versicolor*), show similarly high numbers of protein-coding genes annotated as putative lignin peroxidases, relative to few to no such protein-coding genes in the genomes of either brown-rot fungi (e.g., *Gloeophyllum trabeum*) or soft-rot fungi (e.g., *N. parvum*) [52]. Wood decay in the genus *Fomitiporia* varies from white-rot to brown-rot among species, and some species are not conveniently classified as one or the other type of wood-rotter [53]. Conflicting results from wood-block assays and *in vitro* enzyme-detection assays are not uncommon [50,51], and may reflect the complex structure of woody tissues relative to an artificial medium or the length of the wood-block assay relative to the pace of wood decay. We did not detect manganese peroxidase activity of the *F. polymorpha* isolate we examined, although others have [14]. That said, we used a different assay [45]. One characteristic of Basidiomycetes that are brown-rot or white-rot fungi, which distinguishes them from Ascomycetes that are soft-rot fungi, is the ability to colonize and degrade the middle lamella [51]. From thin sections of the wood blocks, we found only *F. polymorpha* hyphae to colonize the middle lamella, whereas hyphae of the Ascomycetes were absent from this cell layer.

Through a series of three *in vitro* assays, we uncovered novel phenotypic characteristics of *D. seriata* and *N. parvum*: 1. Preferential degradation of pectin (wood block-decay assay), 2. Presence of hyphae in all xylem cell types (wood block-decay assay), 3. Demonstrated activities of pectin lyase, cellulase, hemicellulase, laccase, lignin peroxidase, peroxidase, and polyphenol oxidase (enzyme-detection and quantification assays), 4. No manganese peroxidase activity (enzyme-detection assay), and 5. Growth induction in tannic acid-amended medium (enzyme-detection assay). *Diplodia seriata* and *N. parvum* share these characteristics with other species of Ascomycetes that are soft-rot fungi [50,51]. Stempien et al. [20] report enzyme activities of cellulase, hemicellulase, and laccase from *D. seriata* and *N. parvum*, but their isolates also had manganese peroxidase activity, which was not detected from enzyme-detection assays with our isolates. The *N. parvum*-inoculated wood blocks contained almost twice the percent remaining hemicellulose as the non-inoculated controls. This might be explained as an ‘artifact’ of the method by which hemicellulose was estimated, which was based on the percent remaining holocellulose [39]. High percent remaining holocellulose may reflect depolymerization of cellulose by *N. parvum*, without further breakdown. Regardless, *N. parvum* produced hemicellulase and its growth was induced in hemicellulose-amended medium, which is consistent with activation of *N. parvum* hemicellulase genes *in vitro* and *in planta* [54].

*Diplodia seriata*-inoculated wood blocks had more than two times the percent remaining lignin, compared to that of the non-inoculated controls. This might be explained by the presence of melanin-rich cell walls of *D. seriata* in the extract. Even with filtering, the extract appeared much darker than that of the wood blocks inoculated with the other fungi. That said, advanced soft rot of wood blocks by other Ascomycetes is characterized by a high percentage of remnant lignin, which is the main wood component present after the cellulose, hemicellulose, and pectin are degraded [50]. With no significant differences in percentages of cellulose or hemicellulose remaining of *D. seriata*-inoculated wood blocks, compared to that of the non-inoculated controls, and given that cellulose or hemicellulose are the main components of grape wood [18], it seems more likely that the percentage of lignin remaining was artificially high due to melanin-rich cell walls of *D. seriata* in the extract.

*Eutypa lata* was previously characterized as a soft-rot fungus [16], with preferential degradation of hemicellulose in wood-block assays, and *in vitro* activities of laccase and polyphenol oxidase [18]. We observed the same and additional phenotypic characteristics for our *E. lata* isolate *in vitro*: 1. Preferential degradation of hemicellulose and pectin (wood block-decay assay), 2. Presence of hyphae in all xylem cell types (wood block-decay assay), 3. Demonstrated activities of pectin lyase, cellulase, hemicellulase, laccase, lignin peroxidase, peroxidase, and polyphenol oxidase (enzyme-detection and quantification assays), 4. No manganese peroxidase activity (enzyme-detection assay), and 5. Growth induction in cellulose- or hemicellulose-amended medium (enzyme-quantification assay).

*Neofusicoccum parvum* is more virulent than *D. seriata* and *E. lata*, in terms of rate of lesion formation *in planta* [29,37], and it is possible that its virulence in part reflects its ability to better tolerate and, further, to metabolize phenolic compounds more rapidly. *Neofusicoccum parvum* eliminated the stilbene piceid, and the piceid derivative resveratrol, before *D. seriata* and *E. lata*. *Eutypa lata* was the least tolerant of the three Ascomycetes to resveratrol, as indicated by significant accumulation of resveratrol in piceid-amended medium, before resveratrol was eventually eliminated. Resveratrol dimers, trimers, and tetramers are thought to play a larger role in fungal inhibition than resveratrol, in part based on slower metabolization rates of δ-viniferin (a resveratrol dimer) versus those of resveratrol by *D. seriata* and *N. parvum* [20]. Even with the added difficulty of synthesizing these compounds, more investigation into the breakdown of resveratrol oligomers (and the enzymes activated by the fungi) should be conducted, because measuring the precursor compounds alone may not accurately represent fungal inhibition.

*Neofusicoccum parvum* was the only Ascomycete capable of metabolizing and removing gallic acid from the medium. Gallic acid is the precursor for hydrolysable tannins, which are known to be antimicrobial [55]. Despite strong polyphenol-oxidase activity in wood powder-amended medium, *E. lata* growth was inhibited in gallic acid-amended medium, possibly due to the products of gallic-acid oxidation [56]. Indeed, tannins accumulate in vessel-associated cells of grape wood infected with *E. lata* [19]. Growth of *D. seriata* and *N. parvum* were also inhibited in gallic acid-amended medium, but they had weaker polyphenol-oxidase activity than *E. lata* in wood powder-amended medium. Weak oxidation of gallic acid-amended medium by *D. seriata* and *N. parvum* suggests these fungi do not create an inhospitable environment by extensively oxidizing gallic acid, as *E. lata* does.

Both rutin and epicatechin are flavonoid metabolites that act as antioxidants and have antimicrobial activity [57–60]. Rutin was removed from the medium by the three Ascomycetes in our study. A concomitant accumulation of quercetin (or any derivative) was not detected, indicating that the glycosidic bond between quercetin and rutinoside was not enzymatically hydrolyzed. A lack of quercetin accumulation is consistent with a previous study of rutin degradation by other Ascomycota [61]. Epicatechin metabolization was similar among the three Ascomycetes, but *E. lata* eliminated the most epicatechin (and its derivative) by the ‘mid’ timepoint, despite significant inhibition of its growth by the compound. This is in contrast to a previous report of *E. lata* as more tolerant of epicatechin than both *D. seriata* and *N. parvum* [30]. Flavonoids have been shown to accumulate in grapevine wood during infection by trunk pathogens [29,62,63]. Our findings of flavonoid detoxification by *N. parvum* and growth induction by rutin and epicatechin suggest that such compounds may not inhibit *N. parvum* growth *in planta*.

## Conclusions

‘Endophytes’ is a term used by some (e.g., [64]) to describe a finding of positive detection of the fungi that cause trunk diseases, from a vine with no visible symptoms of trunk diseases. This term seems misleading, given the negative impacts of such fungi on grapevine health. Our findings make it clear that *D. seriata*, *E. lata*, *F. polymorpha*, and *N. parvum* can decay the wood and can use wood components as a source of nutrition, which are actions that negatively affect plant function. Furthermore, a major plant response to infection, production of phenolic compounds, can potentially be ‘undermined’ by detoxification or metabolism of such compounds, especially by *N. parvum*. All four species sporulate on grapevines [23,49,65], and so infected grapevines can potentially serve as a source of inoculum for other grapevines. It is thus important to acknowledge these fungi as pathogens, even at times when infection is not associated with visible symptoms. Given the chronic nature of the wood infections, further work is needed to determine whether removal of infected vines might help limit disease spread within the vineyard.

Now that the types of wood decay caused by *F. polymorpha*, *N. parvum*, and *D. seriata* are known, it may help in the development of new treatments to manage trunk diseases. In order to depolymerize lignin, through a non-enzymatic process known as the ‘chelator-mediated Fenton reaction’, some wood-decay fungi can produce low molecular weight phenolic metabolites, which reduce iron and generate reactive oxygen species [66]. Recent work has demonstrated the possible capacity for non-enzymatic degradation of lignin by *E. lata* [12] and *F. mediterranea* [11]. Furthermore, *in vitro* experiments show that the growth of *E. lata* is inhibited by the antioxidant butylated hydroxy anisole, which either chelates iron and/or prevents the generation of reactive oxygen species [12]. Antioxidants and other compounds with similar activities may, thus, have promise in managing trunk diseases caused by pathogens like *E. lata* and *F. mediterranea*, which are also wood-decay fungi.

## Supporting information

S1 Fig White-rotted wood (see arrow) in cross-section of trunk, of grapevine with leaf symptoms of Esca. *Fomitiporia polymorpha* isolate WFB1 was isolated in culture from the margin of the white-rotted wood and the apparently healthy wood.
(TIF)

S2 Fig Representative color changes in detection of seven enzymes. Enzyme activity was measured by diameter of the color change in the media, relative to colony diameter. Fungi were grown on solid minimal medium amended with each wood component, compared to an unamended control (Enzyme detection, Fig 1A). Detection of manganese peroxidase is not shown because there was no color change for any of the fungi, suggesting that either the assay was not conducive to manganese-peroxidase activity or none of the species tested are white-rot fungi (only white-rot fungi produce manganese peroxidase).
(TIF)
