# Supplementary figures and images for: Biochemical characterization of wood decay and metabolization of phenolic compounds by causal fungi of grapevine trunk diseases
Source: PLoS One. 2025 Apr 16;20(4):e0315412. doi: 10.1371/journal.pone.0315412 (PMC12002536; doi:10.1371/journal.pone.0315412)

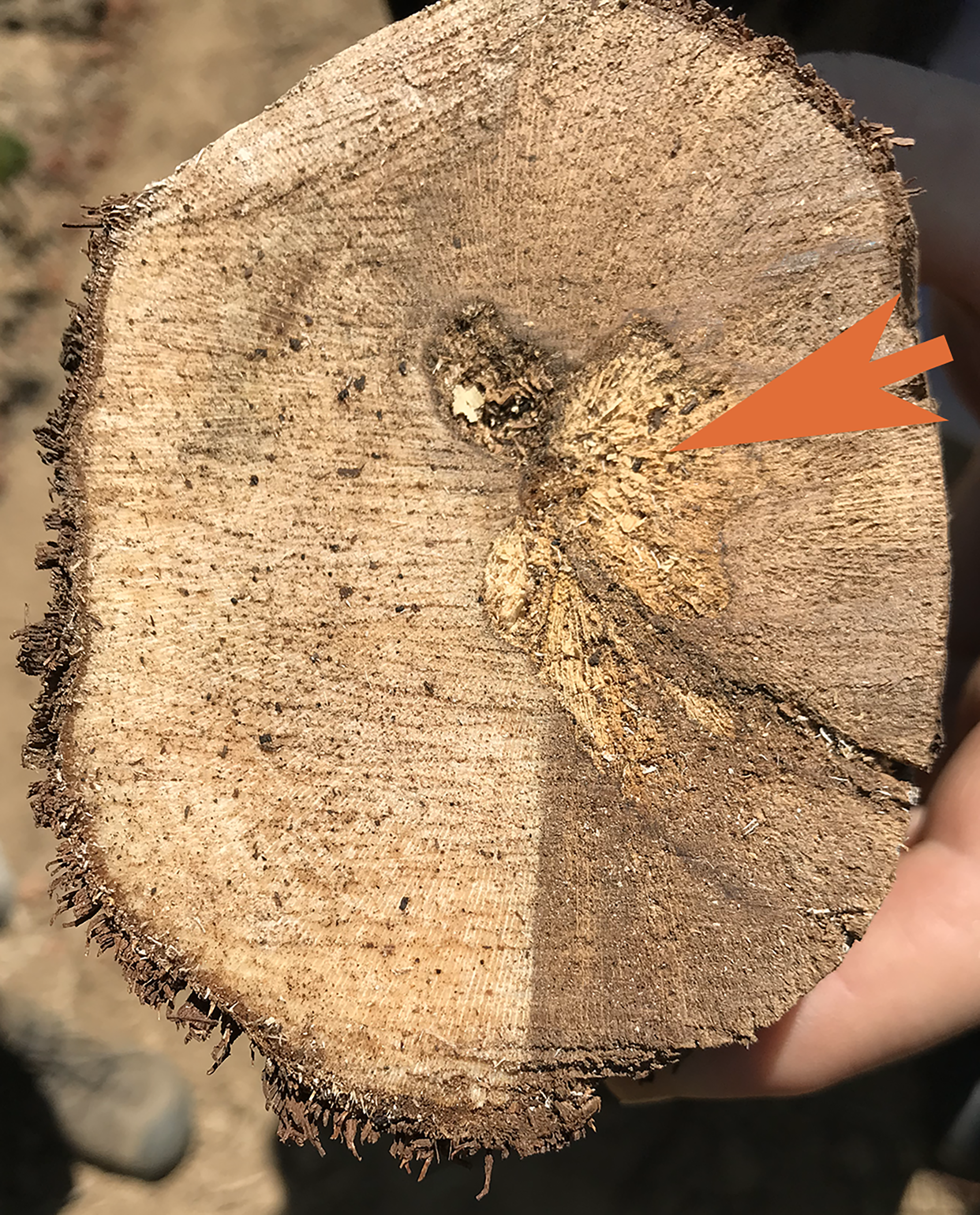

Supplement: S1 Fig — Fomitiporia polymorpha isolate WFB1 was isolated in culture from the margin of the white-rotted wood and the apparently healthy wood. (TIF) [file pone.0315412.s001.tif]

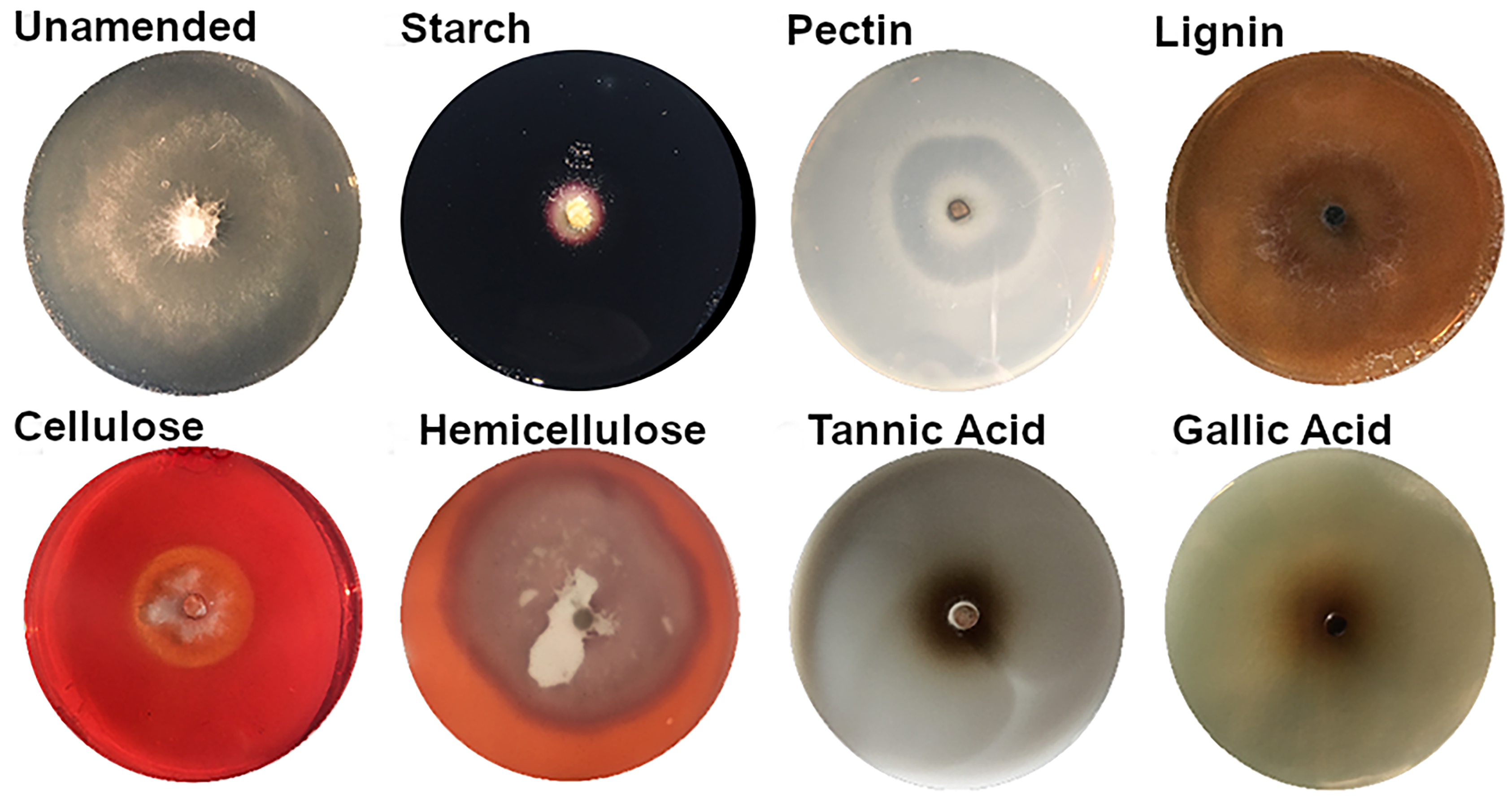

Supplement: S2 Fig — Enzyme activity was measured by diameter of the color change in the media, relative to colony diameter. Fungi were grown on solid minimal medium amended with each wood component, compared to an unamended control (Enzyme detection, Fig 1A). Detection of manganese peroxidase is not shown because there was no color change for any of the fungi, suggesting that either the assay was not conducive to manganese-peroxidase activity or none of the species tested are white-rot fungi (only white-rot fungi produce manganese peroxidase). (TIF) [file pone.0315412.s002.tif]
